# Relationship between the Use of Fentanyl-Based Intravenous Patient-Controlled Analgesia and Clinically Significant Events in Laparoscopic Gynecological Surgery: A Single-Center Retrospective Cohort Study

**DOI:** 10.3390/jcm11113235

**Published:** 2022-06-06

**Authors:** Miho Hamada, Chikashi Takeda, Li Dong, Akiko Hirotsu, Izumi Shizuya, Toshiyuki Mizota

**Affiliations:** Department of Anesthesia, Kyoto University Hospital, Kyoto 606-8507, Japan; isobonger@yahoo.co.jp (M.H.); chikashi@kuhp.kyoto-u.ac.jp (C.T.); dongli@kuhp.kyoto-u.ac.jp (L.D.); akiko162@kuhp.kyoto-u.ac.jp (A.H.); shizuya.izumi@gmail.com (I.S.)

**Keywords:** laparoscopic gynecological surgery, patient-controlled analgesia, postoperative nausea and vomiting, clinically significant event

## Abstract

Background: This study examined the relationship between the use of fentanyl-based intravenous patient-controlled analgesia (ivPCA) and the incidence of a clinically significant event (CSE), while considering both the analgesic effects and side effects in laparoscopic gynecological surgery. Methods: This study included 816 patients undergoing laparoscopic gynecological surgery under general anesthesia at Kyoto University Hospital between 2012 and 2018. The primary exposure was the use of fentanyl-based ivPCA. We defined an outcome measure—CSE—that integrates severe wound pain and vomiting assumed to negatively affect patient recovery. We performed multivariable logistic regression analysis to assess the independent relationship between ivPCA use and CSE. Results: Multivariable logistic regression analysis revealed that fentanyl-based ivPCA was independently associated with increased CSE (adjusted odds ratio (95% confidence interval): 1.80 (1.24–2.61), *p* = 0.002). Use of ivPCA was associated with a reduced incidence of postoperative severe wound pain (adjusted odds ratio (95% confidence interval): 0.50 (0.27–0.90), *p* = 0.022), but was also associated with an increased incidence of vomiting (adjusted odds ratio (95% confidence interval): 2.65 (1.79–3.92), *p* < 0.001). Conclusion: The use of fentanyl-based ivPCA in laparoscopic gynecological surgery is associated with increased CSE.

## 1. Introduction

Although laparoscopic gynecological surgery is known to cause less postoperative pain than open surgery [1,2,3], postoperative pain after laparoscopic gynecological surgery has nevertheless been shown to affect patient recovery [4,5]. Therefore, adequate postoperative analgesia in laparoscopic gynecological surgery is important for promoting patient recovery. A thoracic epidural is not recommended for laparoscopic gynecological surgery [6]; instead, multimodal analgesia with acetaminophen or nonsteroidal anti-inflammatory drugs, in combination with opioids administered orally or with intravenous patient-controlled analgesia (ivPCA), are recommended [6].

Opioids are expected to have a strong analgesic effect, but they are associated with side effects, such as postoperative nausea and vomiting (PONV) and decreased bowel peristalsis [7]. Both PONV and postoperative pain can adversely affect postoperative recovery [8]; thus, even if the administration of opioids reduces postoperative pain, PONV can result in delayed patient recovery. Currently, there is no outcome measure that simultaneously evaluates the analgesic effect and side effects of opioids, making it difficult to assess whether opioid analgesia is beneficial for a patient. We can evaluate whether the use of ivPCA in gynecologic laparoscopic surgery is beneficial to patient recovery only by using an outcome measure that integrates the analgesic effect and side effects, such as PONV, rather than evaluating them separately.

We therefore defined an outcome measure, a clinically significant event (CSE), that integrates severe wound pain and vomiting, assumed to negatively affect patient recovery, and examined the impact of fentanyl-based ivPCA use in laparoscopic gynecological surgery on the incidence of CSE. We hypothesized that the use of fentanyl-based ivPCA would reduce severe wound pain but increase vomiting and consequently increase CSE.

## 2. Materials and Methods

### 2.1. Study Design and Participants

We conducted a single-center retrospective cohort study at Kyoto University Hospital, an 1121-bed teaching hospital in Japan. The study protocol was approved by the Ethics Committee of Kyoto University Hospital (approval number R1272-3), and the requirement for informed consent was waived. This article adheres to the Strengthening the Reporting of Observational Studies in Epidemiology (STROBE) guidelines [9].

Patients who underwent laparoscopic gynecological surgery under general anesthesia (adnexal surgery and/or hysterectomy) at Kyoto University Hospital between January 2012 and April 2018 were eligible for inclusion. We identified eligible patients using the Kyoto University Hospital IMProve Anesthesia Care and ouTcomes (Kyoto-IMPACT) database, which is designed to identify the relationship between intraoperative respiratory and circulatory parameters and postoperative outcomes. We have previously published several papers using this database [10,11,12]. We excluded patients younger than 18 years, patients admitted to the intensive care unit postoperatively, and patients undergoing epidural anesthesia.

### 2.2. Data Collection

We collected the following data from the Kyoto-IMPACT database: patient characteristics (age, height, weight, smoking history, American Society of Anesthesiologists physical status classification, and malignancy) and operative variables (emergency surgery, duration of surgery, intraoperative blood loss, total intravenous anesthesia, total intraoperative fentanyl dose, and intraoperative antiemetic use). In addition, data on the patients’ postoperative courses (the intensity of postoperative wound pain, nausea, and vomiting) were collected from the electronic medical record system. The intensity of postoperative wound pain and the presence or absence of nausea and vomiting were assessed at least twice a day by nurses on the ward. PONV was defined as at least one episode of nausea or vomiting in the two-day postoperative period, and vomiting was defined as at least one episode of vomiting during the same period. Postoperative wound pain was assessed by a six-point Likert-type scale (0 = no pain, 1 = mild pain, 2 = moderate pain, 3 = severe pain, 4 = very severe pain, and 5 = worst possible pain) [13]. Moderate to severe wound pain was defined as the highest pain score recorded in the two postoperative days of 2 (moderate pain) or greater, and severe wound pain was defined as a pain score of 3 (severe pain) or greater.

### 2.3. Exposure

The exposure of interest was the use of fentanyl-based ivPCA. We used a disposable PCA device (COOPDECH Syrinjector^®^; Daiken Medical Co., Ltd., Osaka, Japan) for ivPCA. The PCA device delivers a 1-mL bolus with a lockout interval of 10 min, combined with a baseline infusion of 1 mL/h. In some cases, droperidol was added to the fentanyl-based ivPCA for its antiemetic effect. The decision to use fentanyl-based ivPCA and, if so, the concentration of fentanyl used in the PCA device, as well as whether to add droperidol, was left to the attending anesthesiologist.

### 2.4. Outcomes

The primary outcome was CSE, which was defined as the presence of severe wound pain (pain score ≥ 3 as recorded by the Likert score) or vomiting in the first two postoperative days. We used CSE as the primary outcome because we considered that an integrated measure, rather than separate assessments of analgesic effect and side effects such as PONV, would allow us to evaluate whether the use of fentanyl-based ivPCA in gynecologic laparoscopic surgery is beneficial to patient recovery. Secondary outcomes were postoperative moderate to severe wound pain, postoperative severe wound pain, PONV, and postoperative vomiting.

### 2.5. Statistical Analysis

Continuous variables are presented as median (interquartile range) and compared using the Mann–Whitney U test. Categorical variables are presented as numbers (percentage) and compared using the Pearson chi-square test.

To assess the independent relationship between ivPCA use and CSE, we conducted a multivariable logistic regression analysis to account for the confounding between ivPCA use and CSE. We selected eight potentially confounding variables (age, malignancy, smoking history, total intravenous anesthesia, intraoperative blood loss, duration of surgery, total intraoperative fentanyl dose, and intraoperative antiemetic use) based on the clinical relevance and a literature search for factors that potentially confound the relationship between ivPCA use and CSE [14,15,16,17,18,19]. Each variable included in the logistic regression model demonstrated a variance inflation factor of <10, suggesting no multicollinearity. In addition, patients receiving ivPCA were divided into two groups, according to the baseline infusion rate of fentanyl per kilogram of body weight. We calculated the odds ratio for CSE for each group, with the reference group comprising the patients with no ivPCA use.

We also conducted multivariable logistic regression analyses to assess the relationships between ivPCA use and the following secondary outcomes: postoperative moderate to severe wound pain, postoperative severe wound pain, PONV, and postoperative vomiting. In assessing the relationships between ivPCA use and wound pain, we adjusted for five variables (age, malignancy, operation time, intraoperative blood loss, and intraoperative fentanyl dose) that potentially affected the intensity of wound pain [14]. In assessing the relationships between ivPCA use and PONV and vomiting, we adjusted for four potentially confounding variables (age, smoking history, total fentanyl dose administered intraoperatively, and intraoperative antiemetics use) [15,16,17,19].

Because the relationship between ivPCA use and CSE could depend on patient characteristics or surgical/anesthetic variables, we performed subgroup analyses to assess this potential heterogeneity. The patients were divided into subgroups according to the following factors: age (≥45 years/<45 years); malignancy (yes/no); smoking history (ever smoker/never smoker); duration of surgery (≥240 min/<240 min); total intravenous anesthesia (yes/no); and the use of intraoperative antiemetics (yes/no). The adjusted odds ratio was calculated for each subgroup using the same model as the main analysis, and the interaction between the subgroups and ivPCA use was tested.

To maximize the statistical power, we included in the analysis all eligible patients in the Kyoto-IMPACT database since 2012, when the intensity of postoperative wound pain and nausea/vomiting began to be recorded in its current form. About 120 laparoscopic gynecological surgeries are performed annually at Kyoto University Hospital, so it was estimated that 720 surgeries occurred over a six-year period. We assumed an odds ratio of 1.5, an incidence of CSE of 50%, and a proportion of ivPCA use of 70%, resulting in an estimated power of 98%. As for missing data, we planned to include patients with complete data on variables required for multivariable logistic regression if the proportion of missing data was less than 5%. Such an analysis is feasible in that case [20].

All statistical tests were two-tailed tests, with a *p* value < 0.05 considered statistically significant. All statistical analyses were performed using the statistical program Stata/SE 15.1 (StataCorp LLC^®^, College Station, TX, USA).

## 3. Results

### 3.1. Baseline Patient Characteristics and Operative Variables

**Figure 1:**
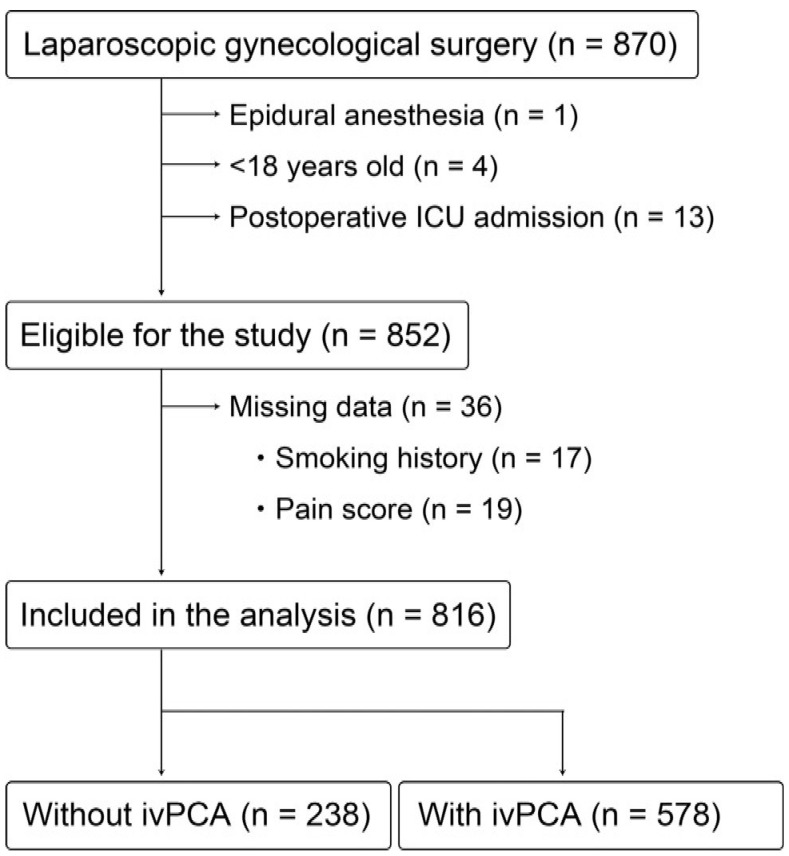
Flow diagram of the study participants. ICU, intensive care unit; ivPCA, intravenous patient-controlled analgesia.

Figure 1 shows the flow diagram of this study. Among 852 patients who were eligible for this study, 36 (4.4%) had missing data on the variables required for multivariable analyses. Fewer than 5% of patients had missing data, so we conducted a complete patient analysis that included 816 patients. Of the 816 study participants, 578 (70.8%) received fentanyl ivPCA. The median baseline infusion rate of fentanyl in patients receiving ivPCA was 0.42 (interquartile range: 0.37–0.48) μg/kg/h. Table 1 shows the patient characteristics and operative variables of the study participants.

### 3.2. Relationship between Intravenous Patient-Controlled Analgesia Use and Outcomes

CSE occurred in 41.2% and 24.8% of patients with and without ivPCA use, respectively (*p* < 0.001). Multivariable logistic regression analysis accounting for potential confounding factors revealed that ivPCA use was independently associated with increased CSE (adjusted odds ratio: 1.80, 95% confidence interval (CI): 1.24–2.61, *p* = 0.002; Table 2).

The incidences of postoperative moderate to severe wound pain, postoperative severe wound pain, PONV, and postoperative vomiting were 48.3%, 7.8%, 59.6%, and 30.9%, respectively. Multivariable logistic regression analyses revealed that ivPCA use was independently associated with reduced incidences of postoperative moderate to severe wound pain (adjusted odds ratio (95% CI): 0.63 (0.45–0.88), *p* = 0.007) and postoperative severe wound pain (adjusted odds ratio (95% CI): 0.50 (0.27–0.90), *p* = 0.022). On the other hand, PONV and vomiting increased in patients with ivPCA use (adjusted odds ratio (95% CI) for PONV: 1.75 (1.27–2.41), *p* = 0.001; adjusted odds ratio (95% CI) for postoperative vomiting: 2.65 (1.79–3.92), *p* < 0.001; Table 3).

We performed an analysis in which the patients receiving ivPCA were divided into two groups according to the baseline infusion rate of fentanyl, and found that CSE tended to increase as the infusion rate of fentanyl increases: the high rate group (fentanyl infusion rate in ivPCA ≥ 0.42 μg/kg/h) had an adjusted odds ratio for CSE of 2.38 (95% CI: 1.58–3.58), whereas the low rate group (fentanyl infusion rate in ivPCA < 0.42 μg/kg/h) had an adjusted odds ratio for CSE of 1.41 (95% CI: 0.92–2.17; Table 4).

**Figure 2:**
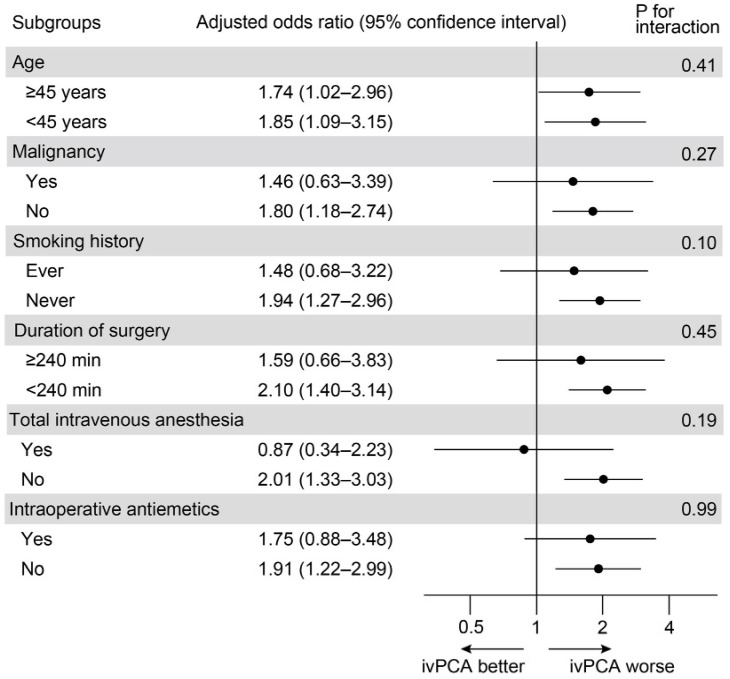
Subgroup analyses stratified by patient and operative variables.

Subgroup analyses based on age, malignancy, smoking history, duration of surgery, total intravenous anesthesia, and the use of intraoperative antiemetics did not significantly affect the relationship between ivPCA use and CSE (Figure 2).

## 4. Discussion

In this study, we revealed that the use of fentanyl-based ivPCA in laparoscopic gynecological surgery was associated with a significant increase in CSE, a composite outcome measure consisting of severe wound pain and vomiting. The use of ivPCA was associated with a decrease in moderate to severe and severe wound pain, but also with a significant increase in PONV and vomiting. In the subgroup analyses, we found no significant influence of patient characteristics or surgical/anesthetic factors on the relationship between ivPCA use and CSE.

We evaluated whether the use of ivPCA in gynecologic laparoscopic surgery is beneficial to patient recovery by using a measure that integrates the analgesic effect and side effects of opioids, rather than evaluating them separately. Despite the fact that patients receiving ivPCA had a significant reduction in severe wound pain, CSE, the primary outcome of this study, increased significantly in patients receiving ivPCA. This increase in CSE in patients receiving ivPCA is presumably attributed to the fact that the increase in vomiting due to ivPCA was greater than the decrease in severe wound pain.

Patients who received high infusion rates (≥0.42 μg/kg/h) of fentanyl in ivPCA tended to develop more vomiting and consequently more CSE as compared with patients who received low infusion rates (<0.42 μg/kg/h), despite a similar frequency of severe wound pain. The infusion rate of fentanyl in ivPCA commonly used in gynecologic laparoscopic surgery is 10 μg/h [21,22,23,24,25], which is calculated to be 0.2 μg/kg/h assuming the patient weighs 50 kg. In light of the above facts, the infusion rates of the high infusion rate group (≥0.42 μg/kg/h) were higher than the commonly used infusion rate. Our data suggest that such a relatively high infusion rate increases CSE and might adversely affect patient recovery. However, in studies of surgeries other than laparoscopic gynecological surgery, a fentanyl-based ivPCA rate of 0.3–0.6 μg/kg/h may be used [26]. Because patient characteristics and the intensity of wound pain vary from procedure to procedure, our results cannot necessarily be extrapolated to other procedures. The impact of ivPCA use on CSE and optimal fentanyl infusion rates in ivPCA should be studied for each procedure.

CSE, the primary outcome of this study, was neither validated nor established as an outcome measure. However, because pain and PONV, which comprise CSE, are common components in validated postoperative recovery measures, such as the postoperative morbidity survey and the postoperative quality of recovery score [27,28,29], these symptoms are considered to be important factors that affect postoperative patient recovery. One advantage of CSE is that it consists of only two components and can be easily evaluated in clinical settings. Future research is needed to verify the validity of CSE.

The incidence of PONV in our study was about 60%, which is similar to or higher than that previously reported in laparoscopic gynecological surgery [21,23,24,25]. Multimodal analgesia and prophylactic antiemetics can be expected to reduce postoperative wound pain, opioid consumption, and PONV. In the future, finding an analgesic regimen that reduces both postoperative wound pain and PONV may be beneficial to patient recovery.

Our study has several limitations. First, the single-center design might limit the generalizability of the results, and external validation is warranted to corroborate our findings. Second, the decision on whether to use fentanyl-based ivPCA, and if so, the fentanyl infusion rate, was left to the attending anesthesiologist. Third, the degree of postoperative wound pain and the incidence of PONV were extracted retrospectively from the electronic medical record system, instead of collected prospectively. For these reasons, this study might suffer from selection bias or information bias. Fourth, although shoulder pain as well as wound pain is an important problem in laparoscopic gynecological surgery, it could not be evaluated in this study because data were not available. Fifth, several potentially important confounding factors, including history of PONV or motion sickness and the use of monitors of anesthesia depth, could not be adjusted, for due to unavailability of data. Finally, the findings are merely an association and cannot imply causation. Thus, we are unable to ascertain whether there is a causal relationship between the use of fentanyl-based ivPCA and increased CSE. Future randomized trials are needed to address this hypothesis.

In conclusion, the use of fentanyl-based ivPCA in laparoscopic gynecological surgery, especially when used at high infusion rates, may increase CSE, the composite outcome measure integrating severe wound pain and vomiting.

## Figures and Tables

**Table 1:** Patient characteristics and operative variables by ivPCA use.

	All Patients (n = 816)	Without ivPCA (n = 238)	With ivPCA (n = 578)	*p* Value
Age (years)	45 (36–56)	40 (32–52)	46 (38–58)	<0.001
Height (cm)	158 (154–162)	158 (154–162)	158.2 (155–162)	0.747
Weight (kg)	53.3 (48.4–59.9)	52.4 (48.1–57.9)	54.0 (48.4–60.7)	0.020
Never smoker	621 (76.1%)	186 (78.2%)	435 (75.3%)	0.379
ASA-PS (1/2/3/missing)	437/360/14/5	132/100/3/3	305/260/11/2	<0.001
Malignancy	223 (27.3%)	38 (16.0%)	185 (32.0%)	<0.001
Emergency surgery	45 (5.5%)	35 (14.7%)	10 (1.7%)	<0.001
Duration of surgery (min)	183 (124–266)	129 (86–186)	212 (148–310)	<0.001
Intraoperative blood loss (mL)	10 (0–100)	0 (0–50)	25 (0–110)	<0.001
Total intraoperative fentanyl dose (μg)	200 (150–250)	150 (100–250)	250 (150–300)	<0.001
Intraoperative antiemetic use	318 (39.0%)	57 (23.9%)	261 (45.2%)	<0.001
Total intravenous anesthesia	144 (17.6%)	50 (21.0%)	94 (16.3%)	0.106

Data were reported as median (interquartile range) for continuous variables and numbers (percentage) for categorical variables. ivPCA, intravenous patient-controlled analgesia; ASA-PS, American Society of Anesthesiologists physical status classification.

**Table 2:** Multivariable logistic regression analysis assessing the independent association between ivPCA use and CSE.

Variable	Adjusted OR (95% CI)	*p* Value
ivPCA	1.80 (1.24–2.61)	0.002
Age (per 10 years)	1.06 (0.95–1.19)	0.295
Malignancy	0.86 (0.56–1.14)	0.474
Duration of surgery (per hour)	1.13 (1.03–1.25)	0.010
Intraoperative blood loss (per mL)	0.99 (0.99–1.00)	0.861
Total intraoperative fentanyl dose (per μg)	0.99 (0.99–1.00)	0.429
Ever smoker	0.81 (0.57–1.14)	0.222
Intraoperative antiemetic use	0.96 (0.70–1.31)	0.800
Total intravenous anesthesia	0.81 (0.54–1.23)	0.328

ivPCA, intravenous patient-controlled analgesia; CSE, clinically significant event; OR, odds ratio; CI, confidence interval.

**Table 3:** Association between ivPCA use and secondary outcomes.

	Event Count		Adjusted OR (95% CI)	*p* Value
	With ivPCA (n = 578)	Without ivPCA (n = 238)		
Postoperative moderate to severe wound pain	266 (46%)	128 (54%)	0.63 (0.45–0.88)	0.007
Postoperative severe wound pain	40 (7%)	24 (10%)	0.50 (0.27–0.90)	0.022
PONV	369 (64%)	117 (49%)	1.75 (1.27–2.41)	0.001
Postoperative vomiting	212 (37%)	40 (17%)	2.65 (1.79–3.92)	<0.001

ivPCA, intravenous patient-controlled analgesia; PONV, postoperative nausea and vomiting; OR, odds ratio; CI, confidence interval.

**Table 4:** Association between fentanyl infusion rate in ivPCA and outcomes.

	Fentanyl Infusion Rate in ivPCA	Adjusted OR (95% CI)	*p* Value
CSE	Without ivPCA	1.00 (reference)	-
	Low rate (<0.42 μg/kg/h)	1.41 (0.92–2.17)	0.070
	High rate (≥0.42 μg/kg/h)	2.38 (1.58–3.58)	<0.001
Postoperative moderate to severe wound pain	Without ivPCA	1.00 (reference)	-
	Low rate (<0.42 μg/kg/h)	0.56 (0.38–0.82)	0.003
	High rate (≥0.42 μg/kg/h)	0.67 (0.46–0.97)	0.034
Postoperative severe wound pain	Without ivPCA	1.00 (reference)	-
	Low rate (<0.42 μg/kg/h)	0.40 (0.20–0.81)	0.010
	High rate (≥0.42 μg/kg/h)	0.54 (0.25–1.05)	0.071
PONV	Without ivPCA	1.00 (reference)	-
	Low rate (<0.42 μg/kg/h)	1.20 (0.84–1.73)	0.316
	High rate (≥0.42 μg/kg/h)	2.56 (1.77–3.72)	<0.001
Postoperative vomiting	Without ivPCA	1.00 (reference)	-
	Low rate (<0.42 μg/kg/h)	2.05 (1.33–3.17)	0.001
	High rate (≥0.42 μg/kg/h)	3.25 (2.14–4.93)	<0.001

ivPCA, intravenous patient-controlled analgesia; CSE, clinically significant event; PONV, postoperative nausea and vomiting; OR, odds ratio; CI, confidence interval.

## Data Availability

Data presented in this study are available upon request from the corresponding authors.
